# Solvation of quantum dots in 1-alkyl-1-methylpyrrolidinium ionic liquids: toward stably luminescent composites

**DOI:** 10.1080/14686996.2020.1735923

**Published:** 2020-03-19

**Authors:** Takuya Nakashima, Kasumi Shigekawa, Shohei Katao, Fumio Asanoma, Tsuyoshi Kawai

**Affiliations:** Division of Materials Science, Nara Institute of Science and Technology, Nara, Japan

**Keywords:** word, nanoparticles, semiconductor, ionic liquid, photoluminescence, solvation, 103 Composites, 204 Optics / Optical applications

## Abstract

CdTe nanoparticles capped with a cationic thiolate ligand were stably dispersed in ionic liquids, 1-alkyl-1-methyl-pyrrolidinium bis(trifluoromethanesulfonyl)amides with an alkyl group of n-propyl, butyl and octyl-chain, and in an ionic plastic crystal, 1-ethyl-1-methylpyrrolidinium bis(trifluoromethanesulfonyl)amide. Dispersion behavior of CdTe nanoparticles in these ionic media was evaluated, in which the solvation of nanoparticles by the ionic components was particularly interested. The ionic media showed alkyl-chain length-dependent solvation behavior, which was suggested by the thermal analysis of nanocomposites. The longer alkyl-chains led to the greater decrease in the thermal melting enthalpy of ionic media with the introduction of nanoparticles. The ionic liquid with an octyl-chain, which is considered to form a thicker solvation layer, afforded better emission durability of CdTe nanoparticles compared to the ionic liquid with a shorter alkyl chain.

## Graphical Abstract

**Figure:**
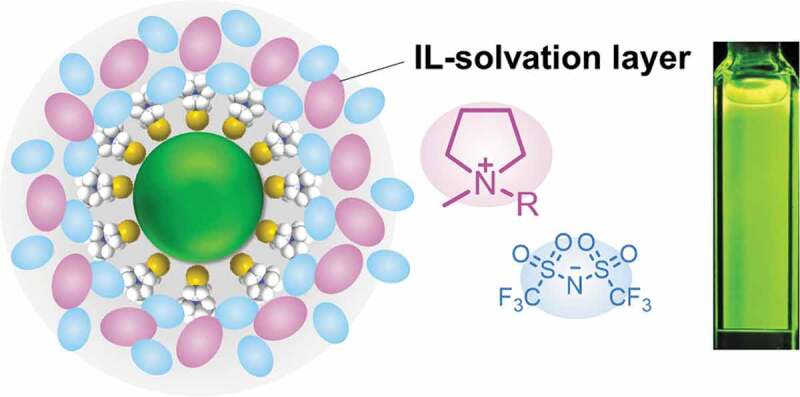

## 1. Introduction

Ionic liquids (ILs) have been studied as media for the synthesis and dispersion of nanoparticles (NPs) [1,2] from viewpoints of their uses in catalysts [3], quasi-solid state electrolytes [4], and optical [5] and luminescent materials [6]. Various bare and ligand-capped NPs were reported to be well stabilized in ILs. The intrinsic self-assembling properties of ILs based on the interionic hydrogen bonding interactions [7,8] and surfactant-like amphiphilic nature [9,10] should support the stabilization of NPs [1,2,11]. The IL-components were presumed to form semi-organized charged supramolecular aggregates to protect bare metallic NPs in steric and electrostatic manners [12]. The formation of solvation layer with an approximate thickness of 5 nm was also suggested for silica particles capped with fluorocarbon molecules by a combination of techniques including dynamic light scattering (DLS) and small-angle neutron scattering (SANS) measurements [13]. A recent work employing atomic pair distribution function (PDF) analysis of X-ray scattering data for an ionic liquid solution of semiconductor NPs unveiled the enhanced ion-ion correlations in the solvation shell [14]. The restructuring of ionic liquid with the oscillatory ion-ion correlations on the NPs was discussed to be responsible for the colloidal stability. The oscillatory distribution of ionic components in a layer-by-layer manner among the solvation layer on the particles was well supported with the surface force measurements and numerical simulations. The surface force measurements by the atomic force microscope (AFM) [15] and by the surface force apparatus (SFA) [16,17] suggested the formation of ordered lamellar-like layer-by-layer structures composed of IL-components on a flat charged surface, which was also supported by a high-energy X-ray reflectivity study [18]. Molecular dynamics (MD) simulations also showed the double-layer formation of IL-components on a negatively charged surface [19].

Surface-capping ligands on NPs further enhance the colloidal stability in ILs especially against temperature and concentration-induced agglomeration [1]. Charged ligands are available to control the colloidal stability of NPs in water in terms of electrostatic stabilization [20–23]. Imidazolium-functionalized surface ligands are often employed to improve the affinity of NP surface to imidazolium-based ILs [24,25]. IL-tethered silica NPs exhibited a good miscibility with a pyrrolidinium-based IL, giving stable composite materials. The strong interaction between the IL-based surface ligand and the IL-components was suggested by a number of measurements. We have recently proposed a cationic capping ligand with a trimethylammonium moiety for a variety of metal and semiconductor NPs with an excellent dispersibility in ILs regardless of the core compositions of NPs [26,27]. Especially semiconductor CdTe NPs capped with thiocholine bis(trifluoromethanesulfonyl)amide (TC-Tf_2_ N) exhibited remarkable stability and improved emission properties in ILs with emission quantum yields higher than 50% [26]. The emission quantum yield of CdTe NPs was further enhanced to almost 100% at temperatures below – 130ºC where the phonon-assisted nonradiative relaxation process is negligible [28]. We attributed the improvement of emission property to the electrostatic stabilization of positively charged capping layer in ILs [26]. The solvation layer by IL-components on the surface should also effectively protect the NP-surface in ILs. However, detailed insights into the solvation of NPs in ILs including the thickness, ordering structure and their dependence on the chemical structure of ILs have not been addressed yet for such the ligand-capped NPs with a single nanometer size. We expect that these characteristics should have a critical impact on the performance of NP-based composite materials. We here study the structure of solvation layer in ILs using the CdTe NPs capped with TC-Tf_2_ N ligand as a model colloid. 1,1-Dialkylpyrrolidinium Tf_2_ N-based ionic compounds with various alkyl-chain lengths including an ionic plastic crystal are delivered as a partner of the NPs. The IL-NPs composites are investigated by means of differential scanning calorimetry (DSC), X-ray diffraction (XRD), ^19^F nuclear magnetic resonance (NMR) and photoluminescence measurements. Finally, the effect of solvation layer is demonstrated as luminescence durability of CdTe NPs under continuous light irradiation.

## 2. Materials and methods

### 2.1. Materials

1-Ethyl-1-methylpyrrolidinium bis(trifluoromethanesulfonyl)amide (P_12_Tf_2_ N), 1-methyl-1-propylpyrrolidinium bis(trifluoromethanesulfonyl)amide (P_13_Tf_2_N), 1-butyl-1-methylpyrrolidinium bis(trifluoromethanesulfonyl)amide (P_14_Tf_2_N) were purchased from Sigma-Aldrich, Kanto-Chemical, and Io-li-tec, respectively. 1-Methyl-1-octylpyrrolidinium bis(trifluoromethanesulfonyl)amide (P_18_Tf_2_N) was synthesized according to the reported procedure [29]. Thiocholine bromide-capped CdTe NPs were prepared according to the previous report [6], followed by anion-exchange with LiTf_2_N in aqueous solution, giving acetone-soluble TC-Tf_2_N capped CdTe NPs [27]. The CdTe NPs-IL nanocomposites were prepared through a co-solvent evaporation method using acetone, since the direct dispersion of NPs resulted in the partial solubilization of NPs in ILs due to the high viscosity of ILs. The mixtures were then dried in a glass tube oven at 80ºC for more than 24 h.

### 2.2. Characterization

Absorption and emission spectra in solution were recorded with a JASCO V-670 spectrophotometer (JASCO, Japan) and a Hitachi F-7000 fluorescence spectrophotometer (Hitachi High-Tech, Japan), respectively. Thermogravimetric analysis (TGA) and differential scanning calorimetry (DSC) were carried out using Shimadzu DTG-60 and DSC-60 (Shimadzu, Japan), respectively. Transmission electron microscopy (TEM) observation was performed with a JEM-2200FS (JEOL, Japan). X-ray diffraction (XRD) patterns were recorded using a Rigaku RINT-TTR III/NM X-ray diffractometer (Rigaku, Japan). XRD peak positions were defined in second derivatives of the measured XRD patterns. ^19^F NMR (376 MHz) spectra were measured with a JEOL ECP-400 spectrometer (JEOL, Japan). A double tube was used. Neat ILs or nanocomposites were placed in the inner tube and the outer tube was filled with CDCl_3_ containing trifluoroacetic acid as an external standard (at – 76.55 ppm). Photo-durability of CdTe NPs under degassed condition were compared in P_13_Tf_2_ N and P_18_Tf_2_N by the irradiation with an Hg-Xe lamp, through a long-pass filter (λ > 440 nm). The intensity of incident light after passing filters was 80 µW cm^−2^ at 440 nm.

## 3. Results and discussion

Water-soluble CdTe NPs capped with thiocholine bromide were first prepared in an aqueous solution [6] and then the bromide anion was exchanged with Tf_2_N using LiTf_2_ N, yielding TC-Tf_2_ N capped NPs [30]. The aqueous CdTe NPs were purified by repetitive precipitation and redispersion processes using methanol and water, respectively, to remove an excess amount of thiocholine bromide prior to the anion-exchange procedure. The CdTe NPs exhibited the first excitonic peak at 540 nm in the absorption spectrum, which led to a NP-size estimation of 3.1 nm (Figures S1 and S2 in the Supplemental material) [31]. TGA showed the mass decrease of NPs by 42 wt% upon heating to 600ºC (Figure S3). The organic (TC-Tf_2_ N ligand) content of 42 wt% corresponds to ca. 100 molecules on a single CdTe core of 3.1 nm. Taking the typical occupied surface area of 22.9 Å^2^ for the thiocholine cation [32] into account the CdTe NPs of 3.1 nm is roughly supposed to support 130 ligand molecules on the surface. The number of ligands given by TGA analysis, therefore, indicates a negligible amount of unbound-free ligand molecules.

**Figure 1.**
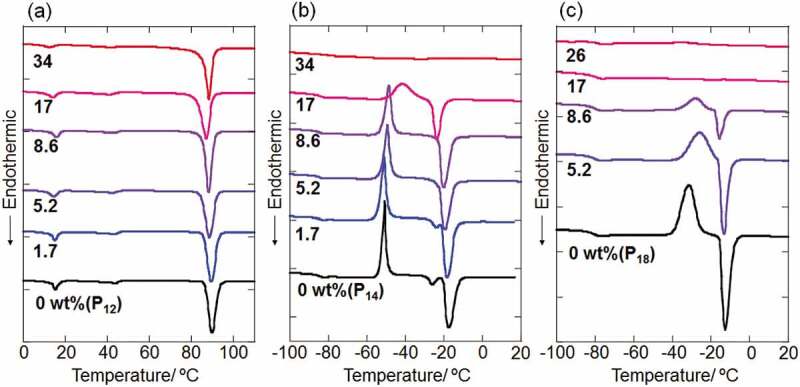
DSC thermograms of IL-CdTe NP composites with (a) P_12_-, (b) P_14_- and (c) P_18_Tf_2_N together with those of 1-alkyl-1-pyrrolidinium salts without NPs

The TC-Tf_2_N capped CdTe NPs were then mixed with 1-alkyl-1-methylpyrrolidinium salts in acetone, which is a good solvent for both components, followed by the complete evaporation of solvent to afford IL-NP composites with various inorganic (CdTe) composition contents. The composites were then evaluated by DSC measurements. Figure 1 shows typical DSC thermograms of composite samples with P_12_Tf_2_N, P_14_Tf_2_N and P_18_Tf_2_N. The y-axes in the DSC charts are normalized with respect to the amount of pyrrolidinium salt in each sample. All the composites of P_12_Tf_2_N gave similar DSC curves regardless of the NPs contents (Figure 1(a)). The thermal transitions at around 15ºC, 45ºC and 90ºC corresponding to phase III–II and II–I transitions and melting, respectively [33], were retained for all samples. Meanwhile, the exothermic and endothermic peaks assigned to cold-crystallization and melting, respectively, decreased with increasing the NPs contents in P_14_- and P_18_Tf_2_N, while the glass transition at around – 80ºC was maintained in all samples (Figure 1(b,c)). The crystallization and melting peaks disappeared by dispersing the NPs with 34 and 17 wt% in P_14_- and P_18_Tf_2_N, respectively. The decrease in the melting endothermic peaks by the introduction of CdTe NPs was also observed for P_13_Tf_2_N samples (Figure S4). The similar behavior was observed for P_14_Tf_2_N composites dispersing SiO_2_ NPs modified with imidazolium ligands [25]. The intercationic interactions mediated by Tf_2_N anion between the cationic NP-surface and pyrrolidinium ions were considered to suppress the crystallization of P_14_Tf_2_N [25]. Such the antifreezing behavior of solvent molecules is often observed for water in the presence of polymers with hydrophilic functional groups [34]. Water molecules strongly bound to the polymers through hydrogen bonding interactions are considered to be in the non-freezable state [34]. It is also interesting to note that the addition of TC-Tf_2_N ligand alone to P_14_Tf_2_N led to small variation in the DSC chart of P_14_Tf_2_N (Figure S5). The introduction of 10 wt% TC-Tf_2_ N salt, which corresponds to the ligand weight fraction in 26 wt% CdTe NPs composites, gave a negligible change in the melting enthalpy (Δ*H*). This result clearly demonstrates that the densely assembled cationic moieties on a nanoparticle configuration play an important role in disturbing the bulk phase behavior of ILs.

**Figure 2.**
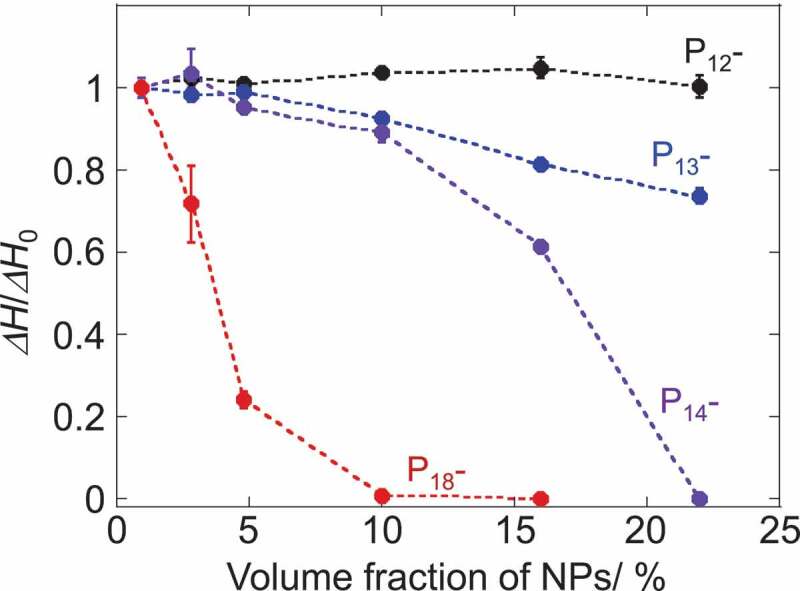
Plots of relative melting enthalpy of 1-alkyl-1-methylpyrrolidinium Tf_2_ N salts as a function of NP-volume fraction. Δ*H*_0_ corresponds to the melting enthalpy of ILs without NPs

**Figure 3.**
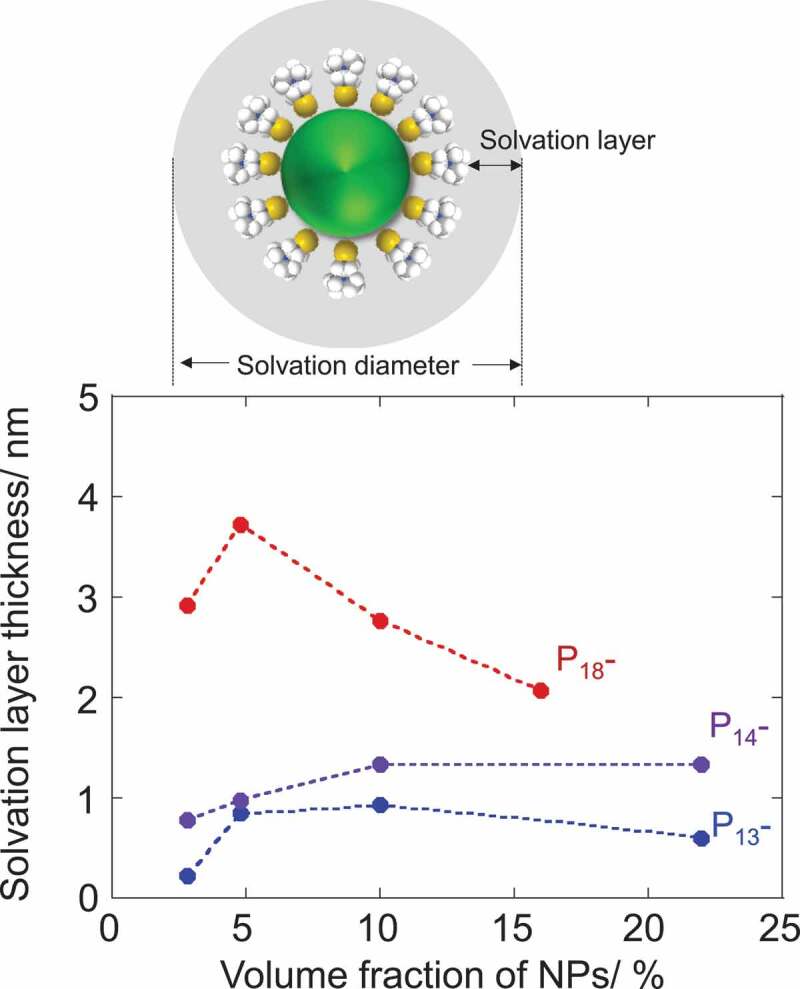
Plots of estimated solvation layer thickness on NPs in 1-alkyl-1-methylpyrrolidinium Tf_2_ N salts as a function of NP-volume fraction together with a schematic representation of solvation layer (above)

The change in the melting enthalpy (Δ*H/*Δ*H*_0_) as a function of the NPs volume fraction was compared between these pyrrolidinium salts (Figure 2). For a clearer comparison, the weight fraction of NPs was converted to the volume fraction (see Table S1 for details). The antifreezing capability of NPs is clearly dependent on the alkyl-chain length in the pyrrolidinium cations of ILs. The melting enthalpy values of P_12_Tf_2_N in the composites were independent of the presence of NPs. The plastic crystal (P_12_Tf_2_N) fraction in the composites showed constant melting behavior regardless of NPs content. This observation was consistent with the previous report on P_12_Tf_2_ N-SiO_2_ NPs mixtures [35]. The interionic interactions between the ionic components in P_12_Tf_2_ N to form and maintain the plastic crystal phase seem stronger than those between the NP-surface and the P_12_Tf_2_ N components. The plastic crystal phase, in which the ionic components are orientationally disordered, may also afford the incorporation of NPs with increasing mean defect size and preserving its plastic crystal phase in bulk [35]. The decrease in the melting enthalpy in the presence of NPs was observed for P_13_-, P_14_- and P_18_Tf_2_N, which exist as liquid at room temperature and do not form the plastic crystal phase. The longer alkyl-chain length led to the more prominent decrease of Δ*H* by the introduction of the same amount NPs. For example, the presence of 10vol% CdTe NPs led to the decrease of Δ*H* by 7, 11 and >99% from the Δ*H*_0_ values for P_13_-, P_14_- and P_18_Tf_2_N, respectively. Given the relative Δ*H/*Δ*H*_0_ value corresponds to the volume fraction of free ILs which behave as ILs in bulk phase unaffected by the NPs, the rest of fraction (1 – Δ*H/*Δ*H*_0_) should be assigned to the IL components interacting (or trapped) with the CdTe NPs in the composites. We here consider that this IL fraction affected by the co-existing NPs should construct the solvation layer on the surface of NPs. Taking this assumption into consideration, we estimated the solvation diameter of NPs in the composites and summarized them in Figure 3 (see Figure S6 and Tables S2–3 in the Supplemental material for details).

The thickness of solvation layer was dependent on the chemical structure of ILs. The longer alkyl chain gave the thicker solvation layer. Meanwhile, it might be mentioned that the thickness of solvation layer should be independent of the NPs concentration in principle. The dynamic nature of trap-release equilibrium of IL components by the NPs may result in the apparently thinner solvation layer values at the low NPs concentration (2.8vol%). The solvation layer thickness was also estimated to decrease above 10vol% of NPs introduction for the composites with P_18_Tf_2_N. According to the DSC study, all the IL components are supposed to be exploited for the solvation in the 10vol% composites. Above this concentration, the solvation layers overlap between NPs and the IL components are shared by a number of NPs, leading to an underestimation of solvation layer thickness over 10vol% NP-contents. P_13_-, P_14_- and P_18_Tf_2_N gave the maximum values of 0.9, 1.3 and 3.7 nm for the solvation layer thickness, respectively. These values further lead to the number of IL-component pairs trapped by the single NP on its surface to be 200, 340 and 1900, respectively, (Tables S2–4). The solvation layer thicknesses estimated are also roughly translated to single, 1–2 and 4–5 ion-pair layers, respectively, which are apparently smaller compared to those measured by the surface force measurements on a flat surface [15–17] but in a similar range to that estimated based on the PDF analysis for NPs in an ionic liquid [14]. The surface force measurement is possible to induce a confinement effect, which enhances the ordering of ionic components confined between two surfaces [16,17]. The surface roughness has also been considered to affect the interfacial ordering of ILs [15,36]. The multifaceted surface of NPs with a single nanometer size cannot serve as a smooth-charged substrate to trigger the strong and long-range organization of IL components in comparison to a flat surface. Nevertheless, the clear dependence of solvation layer thickness on the alkyl-chain length of pyrrolidinium cation suggests a positive correlation of solvation layer thickness with the ordering capability or cohesive energy of IL components in bulk. Self-aggregation of non-polar alkyl groups is more facilitated by the extension of chain lengths via van der Waals forces, leading to the clearer polar-apolar heterogeneity in the microstructure of 1-alkyl-1-methylpyrrolidinium Tf_2_ N ILs with the aid of electrostatic ordering of polar groups [37]. In fact, a prominent first sharp diffraction peak (FSDP), which is attributed to the heterogeneous microstructure, was observed for P_18_Tf_2_N, whereas it was absent for P_13_-, P_14_Tf_2_ N in their X-ray scattering profiles [38].

**Figure 4.**
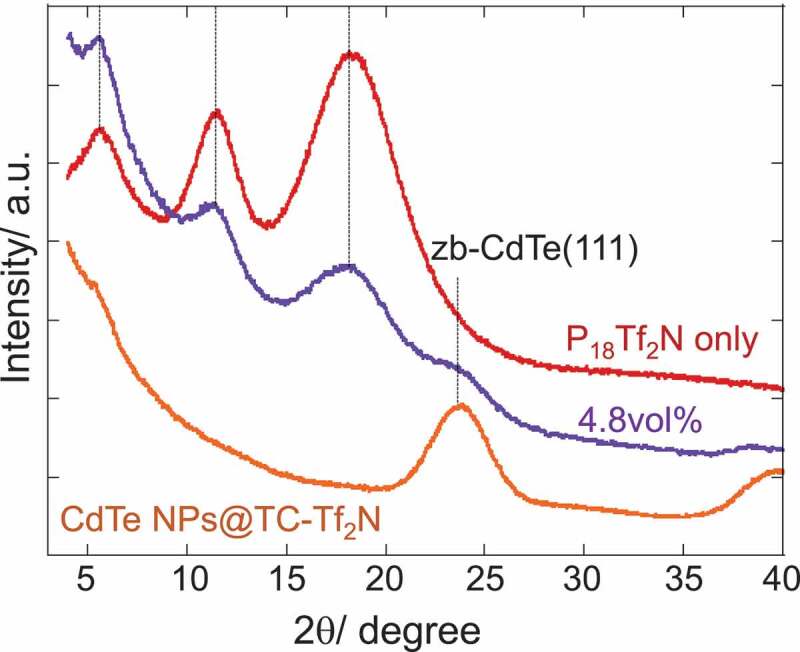
XRD patterns of P_18_Tf_2_N, TC-Tf_2_ N capped CdTe NPs and 4.8vol% composite

An attractive question that is likely to arise is whether the regular layer-by-layer IL-components ordering, which is often depicted as a microstructure of ILs on a charged flat surface, indeed constitutes the solvation layer on the NP-surface. To gain an insight into the detailed structure of the solvation layer, XRD patterns are compared in Figure 4 for P_18_Tf_2_N, TC-Tf_2_ N capped CdTe NPs and their mixture (4.8vol% of NPs). The XRD patterns of P_18_Tf_2_N show three characteristic peaks at 18.3º, 11.8º and 5.8º, providing a good agreement with the reported data [38,39]. Predominant contributors for those peaks were attributed to cation-anion, same-ion correlations and intermediate-range charge ordering, respectively [38,39]. In other words, the peak corresponding to an FSDP at 5.8º is often interpreted as the polar-apolar microphase separation structure [9]. The XRD profile of the composite with 4.8vol% of CdTe NPs shares features both of P_18_Tf_2_N and zincblende(zb)-CdTe. The gradual elevation of background below 20º was observed due to the strong scattering by the dispersed NPs [12]. The 4.8vol%-composite is supposed to lose 74% of bulk property according to the DSC study and this fraction is considered to participate in the solvation structure. However, no apparent peak corresponding to regular lamellar structure appears and three peaks characteristic to P_18_Tf_2_N are still prominent. It is therefore concluded that the IL components gave a limited change from the bulk microstructure to make a solvation layer on the NPs, which could be evaluated as the enhancement of ion-ion correlations by the careful PDF analysis [14]. The orientation of pyrrolidinium cations should change on the NPs but the interionic interactions and van der Waals forces between alkyl chains operate in the solvation layer in a similar range to the bulk phase. The change in the mobility of ionic components may be responsible for the suppression of crystallization in the presence of NPs.

**Figure 5.**
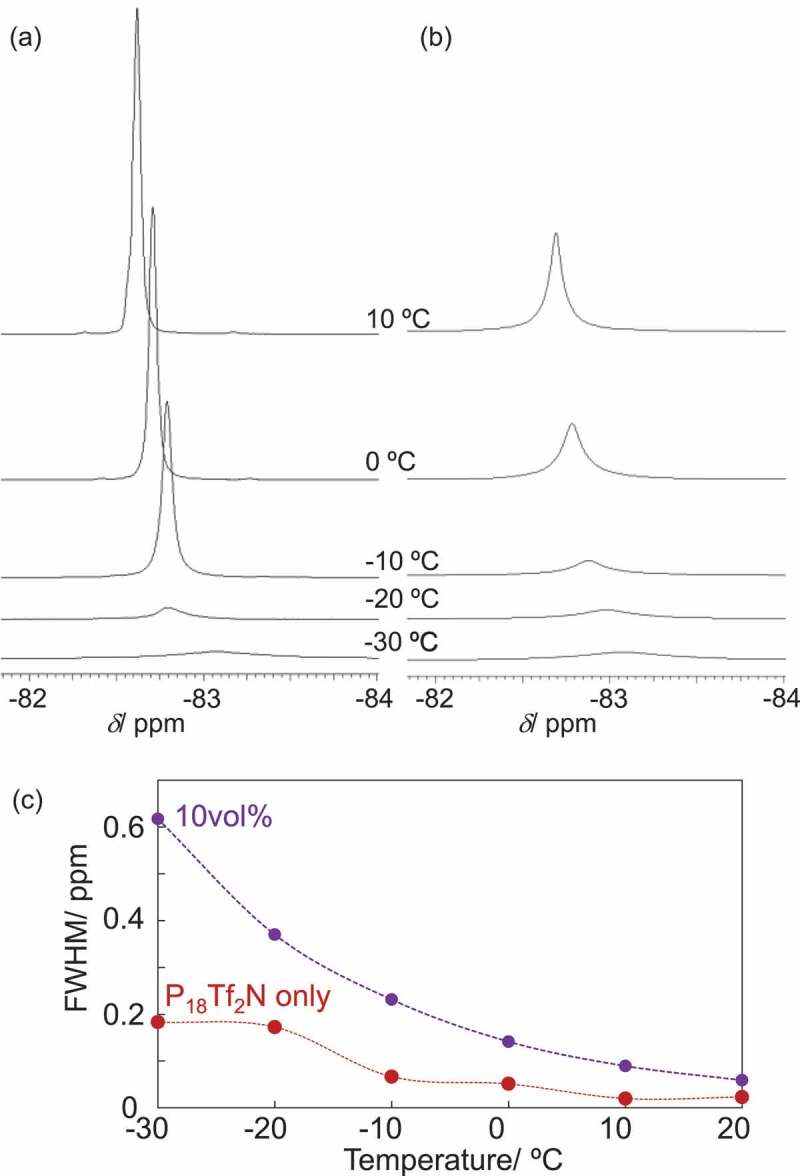
^19^F NMR spectra of (a) P_18_Tf_2_N and (b) its composite with CdTe NPs (10vol%) and (c) their full width at half-maximum (FWHM)

In order to compare the mobility of ionic components in terms of rotational and diffusional motions, temperature-dependent ^19^F NMR measurement was performed [40]. Figure 5 compares the ^19^F peaks corresponding to Tf_2_ N (CF_3_ group) anion in P_18_Tf_2_N and its composite of 10vol%-NPs at different temperatures. Both ^19^F-peaks became sharper upon heating. The full width half maximum (FWHM) values are compared in Figure 5(c). The temperature dependence of FWHM values for P_18_Tf_2_N can be explained by taking the DSC result into account. The IL-components start to crystalize just below – 30ºC and melt at – 13ºC in the absence of NPs, giving a sudden peak sharpening between – 20 and – 10ºC followed by a smooth sharpening above – 10ºC. On the other hand, the composite (10vol%-NPs) did not show such a transition in terms of FWHM temperature dependence, which is in a good agreement with the DSC result showing neither crystallization nor melting transition (Figure 1(c)). Thus, the antifreezing property of the composite was also demonstrated by the ^19^F NMR study. Meanwhile, the FWHM values for the composites are overall larger than those for P_18_Tf_2_N without NPs, clearly supporting the suppressed molecular motion of IL components in the composites. The pyrrolidinium cations should adopt their orientation on the NP-surface with highly oriented and densely packed charges, which propagates over the ion pairs through the cohesive property of IL components, suppressing their reorganization needed in the low-temperature crystallization.

**Figure 6.**
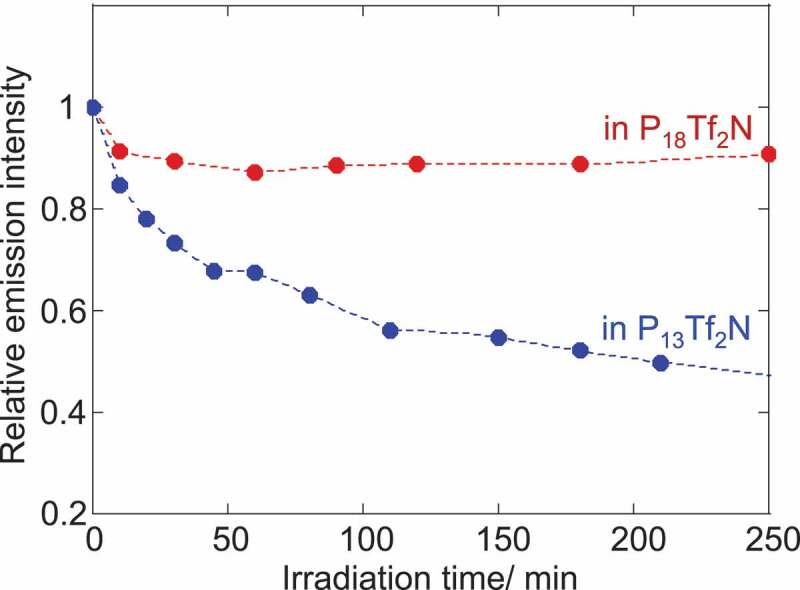
Relative emission intensity of CdTe NPs in ILs under visible irradiation (λ > 440 nm). (NPs concentration: 1.0 × 10^−5^M)

Finally, we have evaluated the effect of the solvation layer on the luminescence of CdTe NPs was evaluated. Their relative emission intensity was compared for two ILs under continuous visible light irradiation in N_2_ atmosphere. Figure 6 clearly demonstrates the dependence of emission photostability on the chemical structure of pyrrolidinium cations. Compared to P_13_Tf_2_ N, P_18_Tf_2_N afforded a higher stability for CdTe NPs. The relative emission intensity of NPs decreased to almost half in P_13_Tf_2_ N after irradiation over 200 min while 90% of initial intensity was maintained in P_18_Tf_2_N. This result agrees with that of solvation layer thickness estimated by the DSC study (Figure 3). There are several possible processes for the photodecomposition of semiconductor NPs [41]. Even though the experiment was conducted under N_2_ atmosphere, the slight amount of residual oxygen can adversely affect the stability of NPs [41]. The photooxidation of NPs leads to the decomposition of semiconductor constituents from the NP-surface. However, the substantial surface passivation with surface ligands effectively protects the NP core from the photooxidation. The IL-components were considered to strengthen such the capping capability of charged ligands on the NP-surface by the electrostatic stabilization and prevent them from desorption processes [6,26,41]. The thicker solvation layer therefore has the better stabilizing capability with the less mobility of IL-components around the NPs.

## 4. Conclusions

We have studied the effect of chemical structure of ILs on the solvation behavior for NPs with charged surface. The interactions of IL-components with the charged NP-surface effectively suppressed the crystallization of IL bulk phase, which was demonstrated by the DSC study. The introduction of longer alkyl chain in the pyrrolidinium cation of ILs enhanced the antifreezing capability of NPs, which was brought about the suppressed mobility of IL-components. While no regular layer-by-layer ordering of IL-components on the NP-surface was suggested for the solvation layer structure, the interionic correlations together with polar-apolar heterogeneity of ILs were found to be preserved in the NPs-IL composites. The CdTe NPs with the thicker solvation layer in the ILs with the longer alkyl-chained pyrrolidinium cations demonstrated the better photostability in terms of emission property. The findings obtained in the present study not only give insights into the solvation mechanism of NPs in ILs but also pave the way for highly luminescent ionic composites of NPs [42] toward emitting devices [43].

## Supplementary Material

Supplemental Material
