# Supplementary material for: Solvation of quantum dots in 1-alkyl-1-methylpyrrolidinium ionic liquids: toward stably luminescent composites
Source: Sci Technol Adv Mater. 2020 Mar 19;21(1):187–94. doi: 10.1080/14686996.2020.1735923 (PMC7144199; doi:10.1080/14686996.2020.1735923)
Supplement: Supplemental Material [file TSTA_A_1735923_SM3875.pdf]

Solvation of quantum dots in 1-alkyl-1-methylpyrrolidinium ionic liquids: toward stably luminescent composites

Takuya Nakashima, Kasumi Shigekawa, Shohei Katao, Fumio Asanoma and Tsuyoshi Kawai*

Graduate School of Materials Science, Nara Institute of Science and Technology, 8916-5 Takayama, Ikoma, Nara 630-0192, Japan

1. Supplementary Figures

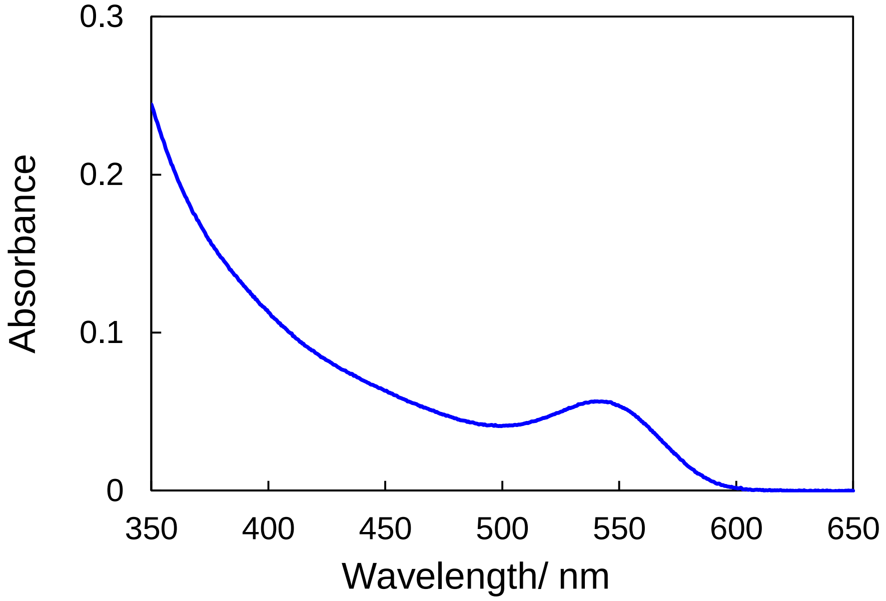

Figure S1. Absorption spectrum of TC-Tf_2N capped CdTe NPs in acetone.

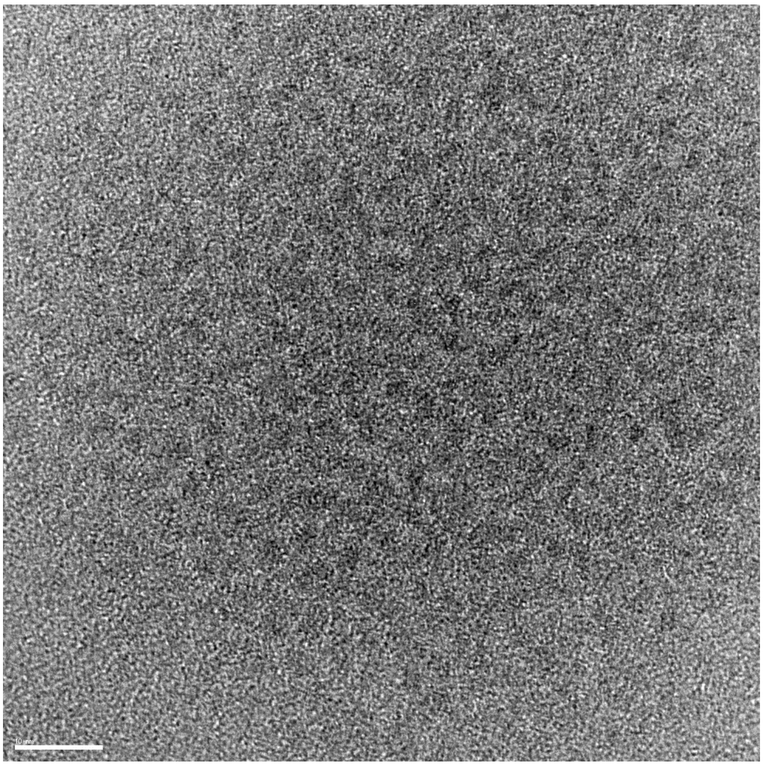

Figure S2. TEM image of TC-Tf_2N capped CdTe NPs.

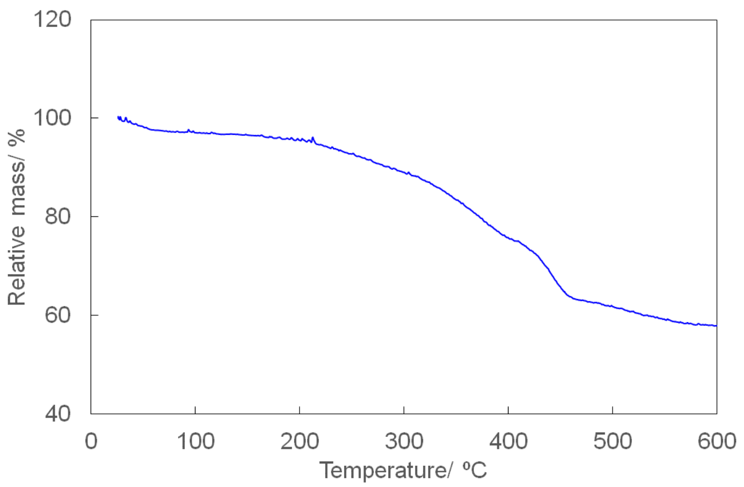

Figure S3. Thermogravimetric curve of TC-Tf_2N capped CdTe NPs.

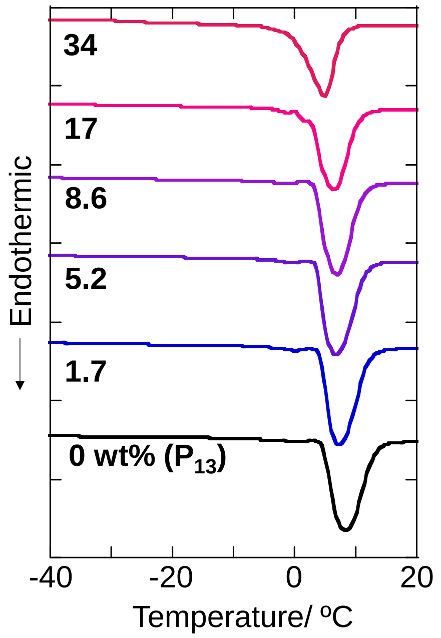

Figure S4. DSC thermograms of CdTe NP composites with $P_{13}Tf_2N$.

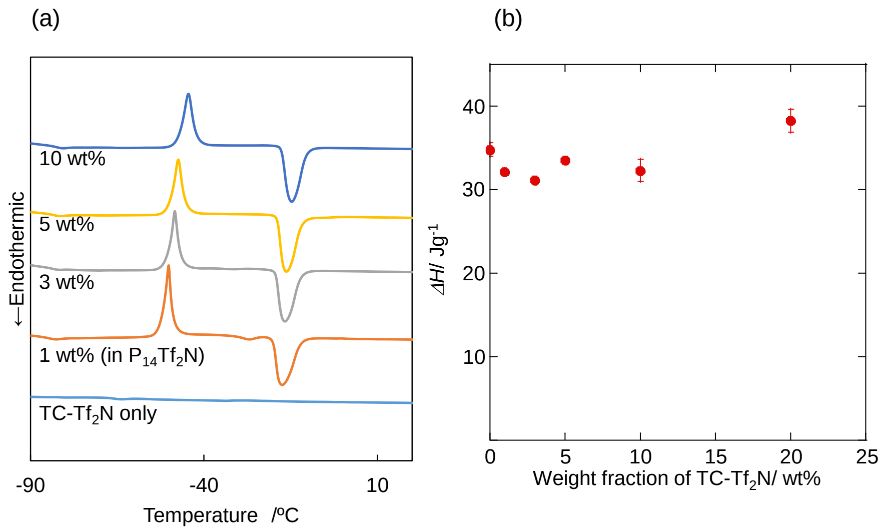

Figure S5. (a) DSC thermograms of $P_{14}Tf_2N$ with $TC\text{-}Tf_2N$ and (b) plot of melting enthalpy (ΔH_m).

2. Estimations of volume fraction of NPs in composites, solvation diamter and number of ion pairs in the solvation layer.

Table S1. Weight and volume fractions of each component in the composites.

$\bar{w}_{CdTe}$ /% (weight fraction of core)	$\bar{w}_{NP}$ /% (weight fraction of NPs)	$\bar{w}_{Lig}$ /% (weight fraction of ligands)	$\bar{w}_{IL}$ /% (weight fraction of ILs)	$\bar{v}_{CdTe}$ /% (volume fraction of core)	$\bar{v}_{Lig}$ /% (volume fraction of ligands)	$\bar{v}_{IL}$ /% (volume fraction of ILs)	$\bar{v}_{NP}$ /% (volume fraction of NPs)
1	1.7	0.72	98	0.17	0.48	70	0.92
3	5.2	2.2	95	0.51	1.45	68	2.8
5	8.6	3.6	91	0.85	2.4	65	4.8
10	17	7.2	82	1.7	2.8	59	10
15	26	11	74	2.6	7.2	53	16
20	34	15	66	3.4	9.7	47	22

$\bar{w}_{NP}$: The inorganic (CdTe) weight fraction of 58%, which was given by TGA measurement, gives the equation as follow: $\bar{w}_{NP} = \bar{w}_{CdTe}/0.58$

$\bar{w}_{Lig}$: $\bar{w}_{Lig} = \bar{w}_{NP} - \bar{w}_{CdTe}$

$\bar{w}_{IL}$: $\bar{w}_{IL} = 100 - \bar{w}_{NP}$

$\bar{v}_{CdTe}$: $\bar{v}_{CdTe} = \bar{w}_{CdTe}/5.855$ (density of CdTe = 5.855 gcm^{-3})

$\bar{v}_{Lig}$: $\bar{v}_{Lig} = \bar{w}_{Lig}/1.5$ (We assume the density of ligand layer as 1.5 gcm^{-3}.)

$\bar{v}_{IL}$: $\bar{v}_{IL} = \bar{w}_{IL}/1.4$ (We assume the density of ILs as 1.4 gcm^{-3}.)

$\bar{v}_{NP}$: $\bar{v}_{NP} = (\bar{v}_{CdTe} + \bar{v}_{Lig})/(\bar{v}_{CdTe} + \bar{v}_{Lig} + \bar{v}_{IL})$

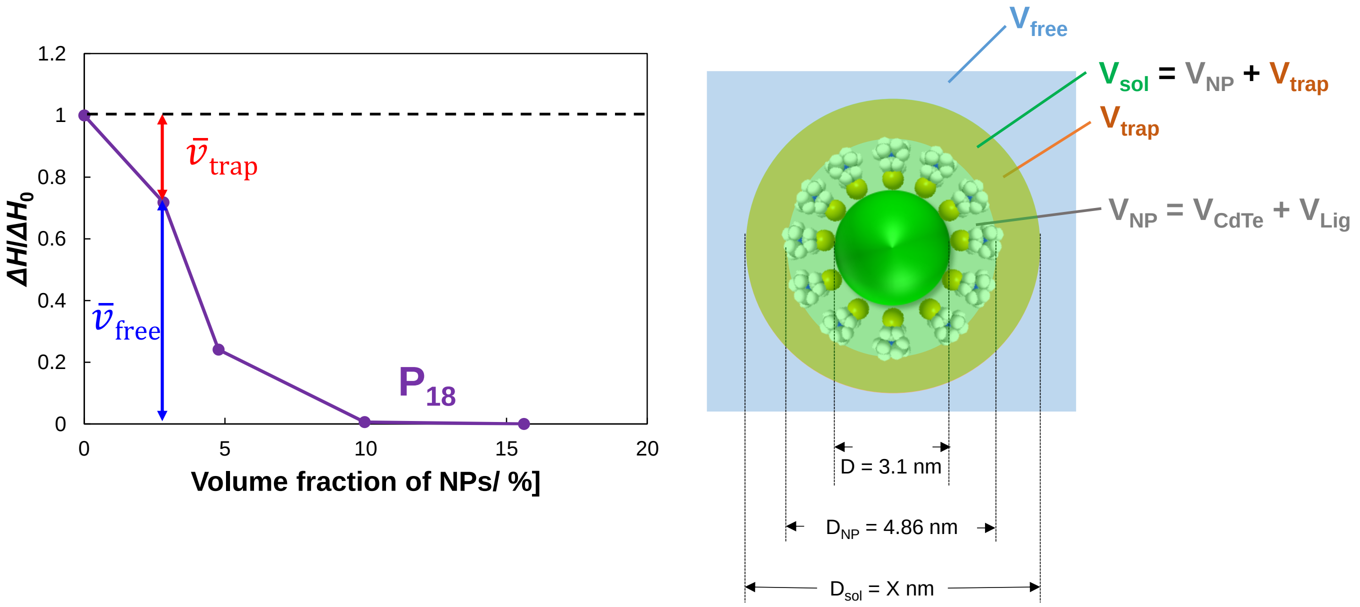

Figure S6. Schematic representation of the solvation layer of NPs.

CdTe core; diameter: D = 3.1 nm, volume: $V_{\mathrm{CdTe}} = 1.56 \times 10^{-20}$ cm^3, density: $d_{\mathrm{CdTe}} = 5.855$ gcm^{-3}, weight of single core: $W_{\mathrm{CdTe}} = 9.13 \times 10^{-20}$ g

The inorganic (CdTe) weight fraction of 58%, which was given by TGA measurement, led to the weight of a single NP (W_{NP}) including the weight of ligand layer (W_{Lig}).

$W_{\mathrm{NP}} = W_{\mathrm{CdTe}}/0.58 = 1.57 \times 10^{-19}$ g, $W_{\mathrm{Lig}} = W_{\mathrm{NP}} - W_{\mathrm{CdTe}} = 6.61 \times 10^{-20}$ g

Given the ligand (TC-$\mathrm{Tf_2N}$) density of ca. 1.5 gcm^{-3}, the volume of ligand layer (V_{Lig}) in the single NP was estimated as follows.

$V_{\mathrm{Lig}} = W_{\mathrm{Lig}}/1.5 = 4.41 \times 10^{-20}$ cm^3, then $V_{\mathrm{NP}} = V_{\mathrm{CdTe}} + V_{\mathrm{Lig}} = 5.97 \times 10^{-20}$ cm^3

$V_{\mathrm{CdTe}}/V_{\mathrm{NP}} = 26$ vol% in the single particle.

If we assume the spherical shape for the NPs, the radius of NP including the ligand layer (r_{NP}) was given as $r_{\mathrm{NP}} = 2.43$ nm (diameter of NP: $D_{\mathrm{NP}} = 4.86$ nm).

Table S2. Estimations solvation diameter and number of ion pairs interacting with single NP in $P_{13}Tf_2N$.

$\bar{v}_{NP}$ /% (volume fraction of NPs)	$\Delta H/\Delta H_0$	$\bar{v}_{trap}$ /% (volume fraction of trapped ILs)	D_{sol}/ nm (diameter of solvation layer including D_{NP})	V_{trap}/ cm^3 (volume of solvation layer without V_{NP})	W_{trap}/ g (weithg of solvation layer without V_{NP})	N_{trap} (numer of ion pairs interacting with single NP)
2.8	0.99	1.0	5.32	1.9×10^{-20}	2.7×10^{-20}	40
4.8	0.93	7.0	6.55	8.7×10^{-20}	12×10^{-20}	180
10	0.81	16	6.72	9.9×10^{-20}	14×10^{-20}	200
22	0.74	21	6.06	5.7×10^{-20}	7.9×10^{-20}	120

Table S3. Estimations solvation diameter and number of ion pairs interacting with single NP in $P_{14}Tf_2N$.

$\bar{v}_{NP}$ /% (volume fraction of NPs)	$\Delta H/\Delta H_0$	$\bar{v}_{trap}$ /% (volume fraction of trapped ILs)	D_{sol}/ nm (diameter of solvation layer including D_{NP})	V_{trap}/ cm^3 (volume of solvation layer without V_{NP})	W_{trap}/ g (weithg of solvation layer without V_{NP})	N_{trap} (numer of ion pairs interacting with single NP)
2.8	0.95	4.6	6.42	7.8×10^{-20}	1.1×10^{-19}	160
4.8	0.89	10	6.80	1.0×10^{-19}	1.5×10^{-19}	220
10	0.62	33	7.54	1.6×10^{-19}	2.3×10^{-19}	340
22	0	78	7.52	1.6×10^{-19}	2.3×10^{-19}	340

Table S4. Estimations solvation diameter and number of ion pairs interacting with single NP in $P_{18}Tf_2N$.

$\bar{v}_{NP}$ /% (volume fraction of NPs)	$\Delta H/\Delta H_0$	$\bar{v}_{trap}$ /% (volume fraction of trapped ILs)	D_{sol}/ nm (diameter of solvation layer including D_{NP})	V_{trap}/ cm^3 (volume of solvation layer without V_{NP})	W_{trap}/ g (weithg of solvation layer without V_{NP})	N_{trap} (numer of ion pairs interacting with single NP)
2.8	0.72	27	10.7	5.9×10^{-19}	8.2×10^{-19}	1200
4.8	0.24	72	12.3	9.1×10^{-19}	13×10^{-19}	1900
10	0.006	89	10.4	5.4×10^{-19}	7.6×10^{-19}	1100
16	0	84	9.0	3.2×10^{-19}	4.5×10^{-19}	670

$\bar{v}_{trap} = \bar{v}_{IL} \times (1 - \Delta H/\Delta H_0)$: volume fraction of IL interacting with NPs (non-freezing ILs)

V_{sol} and V_{trap} respresents a solvation volume with and without the volume of NP (V_{NP}), respectively.

$$V_{trap}/V_{NP} = (V_{sol} - V_{NP})/V_{NP} = \bar{v}_{trap}/\bar{v}_{NP}$$

$$\frac{3}{4}\pi\left(\frac{D_{sol}}{2}\right)^3 - \frac{3}{4}\pi\left(\frac{D_{NP}}{2}\right)^3 = \frac{3}{4}\pi\left(\frac{D_{NP}}{2}\right)^3 \times \bar{v}_{trap}/\bar{v}_{NP}$$

$$\left(\frac{D_{sol}}{2}\right)^3 = \left(\frac{D_{NP}}{2}\right)^3 + \left(\frac{D_{NP}}{2}\right)^3 \times \bar{v}_{trap}/\bar{v}_{NP}$$

$$V_{trap} = V_{NP} \times \bar{v}_{trap}/\bar{v}_{NP} = 5.97 \times 10^{-20} \times \bar{v}_{trap}/\bar{v}_{NP}\ cm^3$$

$W_{trap} = V_{trap} \times 1.4$ ($d_{IL} = 1.4\ gcm^{-3}$): Weight of IL components interacting with single NP

$N_{trap} = W_{trap}$ /Mw × N_A (Mw: molecular weithg of IL, N_A: Avogadro number)
